# Development of Pectinase Based Nanocatalyst by Immobilization of Pectinase on Magnetic Iron Oxide Nanoparticles Using Glutaraldehyde as Crosslinking Agent

**DOI:** 10.3390/molecules28010404

**Published:** 2023-01-03

**Authors:** Tayyaba Behram, Sidra Pervez, Muhammad Asif Nawaz, Shujaat Ahmad, Amin Ullah Jan, Haneef Ur Rehman, Shahbaz Ahmad, Nasir Mehmood Khan, Farman Ali Khan

**Affiliations:** 1 Department of Biotechnology, Shaheed Benazir Bhutto University, Sheringal Dir Upper 18000, Pakistan; 2 Department of Biochemistry, Shaheed Benazir Bhutto Women University, Peshawar 25000, Pakistan; 3 Department of Pharmacy, Shaheed Benazir Bhutto University, Sheringal Dir Upper 18000, Pakistan; 4 Department of Natural and Basic Sciences, University of Turbat, Kech, Turbat 92600, Pakistan; 5 Department of Biological Science and Engineering, School of Chemistry and Biological Engineering, University of Science and Technology Beijing, Beijing 100083, China; 6 Department of Agriculture, Shaheed Benazir Bhutto University, Sheringal Dir Upper 18000, Pakistan; 7 Department of Chemistry, Shaheed Benazir Bhutto University, Sheringal Dir Upper 18000, Pakistan

**Keywords:** pectinase, *Bacillus subtilis*, immobilization, iron oxide nanocarrier

## Abstract

To increase its operational stability and ongoing reusability, *B. subtilis* pectinase was immobilized on iron oxide nanocarrier. Through co-precipitation, magnetic iron oxide nanoparticles were synthesized. Scanning electron microscopy (SEM) and energy dispersive electron microscopy (EDEX) were used to analyze the nanoparticles. Pectinase was immobilized using glutaraldehyde as a crosslinking agent on iron oxide nanocarrier. In comparison to free pectinase, immobilized pectinase demonstrated higher enzymatic activity at a variety of temperatures and pH levels. Immobilization also boosted pectinase’s catalytic stability. After 120 h of pre-incubation at 50 °C, immobilized pectinase maintained more than 90% of its initial activity due to the iron oxide nanocarrier, which improved the thermal stability of pectinase at various temperatures. Following 15 repetitions of enzymatic reactions, immobilized pectinase still exhibited 90% of its initial activity. According to the results, pectinase’s catalytic capabilities were enhanced by its immobilization on iron oxide nanocarrier, making it economically suitable for industrial use.

## 1. Introduction

Pectinase is a group of heterogeneous enzymatic reactions for the degradation of pectin and pectin-containing compounds. Pectinases are used in a variety of industrial processes, including fruit juice extraction, textile processing and bioscouring of cotton fibers, degumming of remain, retting of flax, coffee and tea fermentation, oil extraction, plant protoplast formation, and pretreatment of pectic waste during water treatment [1,2,3]. In addition to these diverse uses, the industrial use of pectinases is hampered by their low thermal, storage, and operational stabilities [4,5]. Immobilization is a method that not only enhances the catalytic performance of biocatalysts but also increases their operational stability and makes them reusable for ongoing industrial processes [5].

There are several uses for immobilized biocatalysts in industrial and commercial processes. These techniques provide greater operational cycle control, resulting in a more flexible reactor design. The separation of immobilized biocatalysts from the process is a simple and cost-effective method for downstream product purification [6].

There are numerous methods for immobilizing enzymes. Each method has its own benefits and drawbacks. By optimizing several circumstances for a given enzyme, it is possible to select an appropriate technique. Immobilization techniques include adsorption, entrapment, covalent bonding, and cross-linking. The adsorption and entrapment processes are easier, but weak contacts cause enzyme leaching. However, covalent binding and cross-linking produce strong interactions, but they can alter the shape of enzyme structures, resulting in enzyme inactivation [7]. In the past, entrapment, adsorption, and covalent binding have been used to immobilize pectinase [4,8,9]. In addition, the selection of a support system for the immobilization process is crucial, as the features of the support impact the overall capability of an immobilized enzyme reaction [10].

There have been reports of the immobilization of pectinase using a variety of support materials, including alginate beads [4], agar gel [5], and polyacrylamide gel [11,12]. Nanocarriers are frequently used as support materials for the immobilization of the enzyme due to their unique qualities, including high surface area, shape retention, availability in diverse sizes and compositions, and characteristics after functional activation of the surface [13,14,15]. Several methods for immobilizing the enzyme have been developed, but effective support materials and immobilization methods are currently being explored to overcome difficulties associated with commercial usage of the pure enzyme [16]. In addition to the choice of immobilization technology, the selection of an adequate matrix for the immobilization procedure is crucial. The selection of matrix depends on the enzyme of interest as well as certain matrix features, such as appropriate mechanical stability and nontoxicity for immobilized biocatalysts, cells, and the product formed [7].

Due to their chemical, physical, and superparamagnetic properties, as well as their high surface area for reactivity, magnetic nanoparticles (MNPs) have been deemed promising carriers for immobilizing enzymes [17,18,19]. The superparamagnetic of MNPs makes it simple to remove the immobilized enzyme from the reaction medium using a magnetic field. In comparison to polymeric porous materials, the nanoscale characteristics of MNPs carriers give a large specific surface area for increased enzyme reactivities and reduce or limit the steric effect of impediment [19]. Iron oxide nanoparticles are a cost-effective and acceptable solid carrier for enzyme immobilization [19]; their synthesis is reasonably inexpensive. In the present study, pectinase was immobilized on functionally activated iron oxide nanoparticles to obtain great stability and reusability. For maximal immobilization yields and catalytic activity, the immobilization parameters and immobilized iron oxide nanoparticle pectinase were optimized and characterized. In addition, the reusability of immobilized pectinase for industrial applications was analyzed.

## 2. Results and Discussion

### 2.1. Preparation of Magnetic Iron Oxide Nanoparticles

The nanoparticles were synthesized using the co-precipitation process. This is the simplest and most efficient approach for synthesizing metal oxide nanoparticles. In this approach, stoichiometric quantities of metal salts in proper molar ratios are introduced to basic media (pH 8–14), resulting in precipitate formation. The magnetic nanoparticles were synthesized using a 2:1 Fe^+3^/Fe^+2^ ratio. Equation (1) describes the reaction process in detail.

(1) $${\mathrm{Fe}}^{+3}+{\;\mathrm{Fe}}^{+2}\overset{8{\mathrm{OH}}^{-}}{\to }{\mathrm{Fe}}_{3}O_{4}+4H_{2}O$$

As Fe_3_O_4_ is prone to oxidation and is extremely unstable, it is further oxidized in the presence of air through the calcination process, resulting in the formation of stable γ-Fe_2_O_3_ (Equation (2)).

(2) $${\mathrm{Fe}}_{3}O_{4}+2H^{+}\to \gamma –{\mathrm{Fe}}_{2}O_{3}+{\;\mathrm{Fe}}^{+2}+{\;H}_{2}O$$

**Figure 1:**
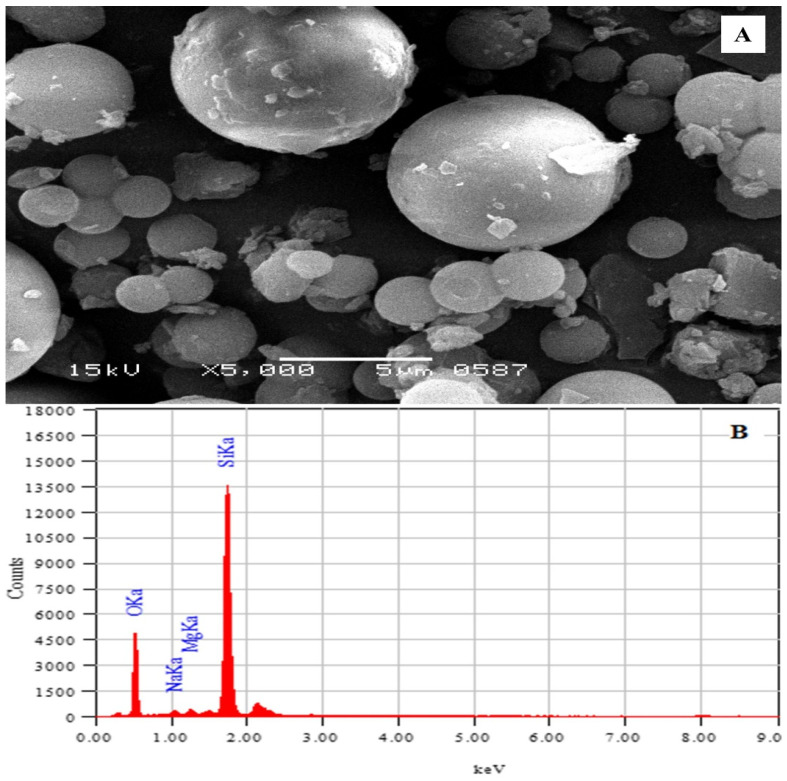
(**A**) Scanning electron image; (**B**) EDAX spectra of magnetic iron oxide nanoparticles.

Scanning electron microscopy (SEM) and energy dispersive electron microscopy (EDEM) were used to characterize the nanoparticles that were produced (EDAX). The particle size and surface morphology of iron oxide nanoparticles were investigated using SEM. The electron micrograph of iron nanoparticles is shown in Figure 1A. The data clearly shows that the particle size of produced iron oxide particles is in the nanometer range. The scale indicates that the particle size is less than 100 nm, with a narrow particle size distribution. It is also clear that the particles did not agglomerate, confirming the particles’ superior dispersion capacity. This property was highly useful for improving the immobilization capabilities of the produced nanoparticles and yielded better results than earlier research. EDAX spectra of magnetic iron oxide nanoparticles are shown in Figure 1B. The graphic clearly shows that all of the elements of iron oxide are present at their relevant energy level. Peaks arise at 6.5 and 7.1 KeV and are indicative of Fe, whereas a high-intensity peak at 0.5 KeV is suggestive of oxygen. The iron mass percent in the fabricated nanoparticle is predicted to be 47.91%, whereas the oxygen mass percent is 39.75%. These findings validated the existence and proportion of both components in produced nanoparticles. Furthermore, employing glutaraldehyde as a crosslinking agent, the partially purified pectinase was immobilized on magnetic iron oxide nanoparticles. The aldehydic groups of aldehyde are particularly reactive in terms of forming covalent bonds with various functional groups such as amines, thiols, phenols, hydroxyls, and imidazole’s and they may be involved in the covalently binding of the enzyme to magnetic iron nanoparticles.

### 2.2. Optimization of Immobilization Parameters of Pectinase on Magnetic Iron Nanoparticles

#### 2.2.1. Effect of Crosslinking Concentration

**Figure 2:**
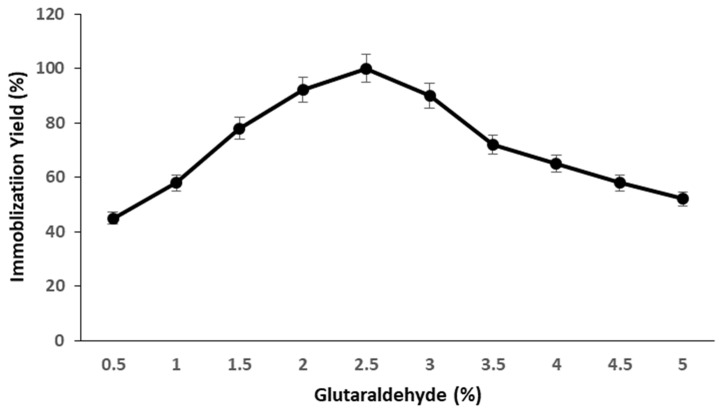
Effect of glutaraldehyde concentration on the immobilization of pectinase on magnetic iron oxide nanocarrier.

Glutaraldehyde often crosslinks the enzyme through intra- and intermolecular cross-linking formation reactions. While a low dosage of glutaraldehyde may not be sufficient to bind a maximum concentration of enzyme with maximum enzymatic activity, a high concentration of the crosslinking agent may alter the structure of enzymes, rendering them inert. As a result, the impact of glutaraldehyde concentration on pectinase immobilization in terms of maximal enzymatic retention was investigated using different concentrations of glutaraldehyde ranging from 0.5% to 5.0%. (Figure 2). The residual activity of pectinase increased with glutaraldehyde concentration, and the maximum residual activity was obtained when 2.5% glutaraldehyde concentration was employed in the immobilization technique. Increased glutaraldehyde concentration reduced pectinase residual activity, and pectinase lost 48% of its residual activity at a concentration of 5.0%. Excess glutaraldehyde may induce unfavorable chemical changes in the enzyme, deactivating its 3D structure for catalytic activity. A high concentration of glutaraldehyde may also block the active side of enzymes, compromising substrate binding and decreasing enzyme catalytic activity.

#### 2.2.2. Effect of Enzyme Concentration

**Figure 3:**
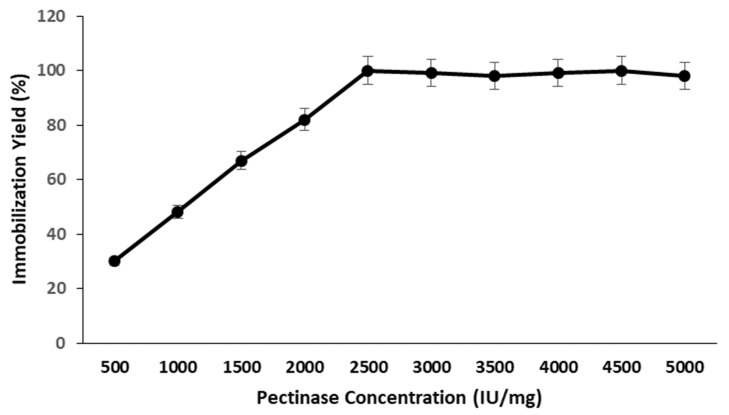
Effect of enzyme concentration on the immobilization of pectinase on magnetic iron oxide nanocarrier.

The optimal enzyme concentration is critical for retaining maximum residual activity following immobilization. During the immobilization technique, the concentration of pectinase was optimized for optimal residual activity by utilizing several concentrations of enzyme solution ranging from 500 to 5000 IU/mg. The residual activity of pectinase immobilized magnetic iron oxide nanocarrier increased with increasing enzyme concentration, with 2500 IU/mg exhibiting the highest residual activity. The residual activity of immobilized pectinase achieved saturation at a concentration of 2500 IU/mg, and the residual activity of nanocarrier immobilized pectinase remained unchanged when the enzyme concentration was raised further (Figure 3).

#### 2.2.3. Effect of Crosslinking Time

**Figure 4:**
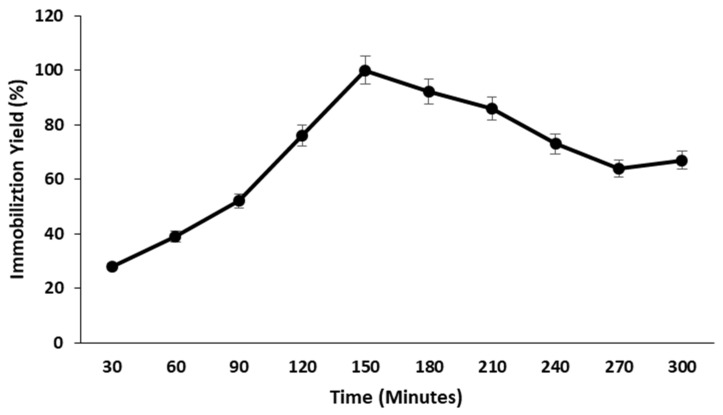
Effect of crosslinking time on the magnetic iron oxide nanocarrier.

The impact of crosslinking duration on pectinase immobilization on nanocarrier was investigated by reacting glutaraldehyde solution with nanocarrier for various time periods ranging from 30 to 300 min. The maximal residual activity of immobilized pectinase was observed after 150 min of reaction time, and after this time period, the residual activity of nanocarrier-immobilized pectinase decreased (Figure 4). The shorter the crosslinking time, the less likely the crosslinking agent will bind the nanocarrier to the maximum enzyme attached, and the longer the crosslinking time, the greater the steric hindrance impact on enzyme molecules and the restriction of substrate binding to the active side of enzymes.

#### 2.2.4. Effect of Immobilization Temperature

**Figure 5:**
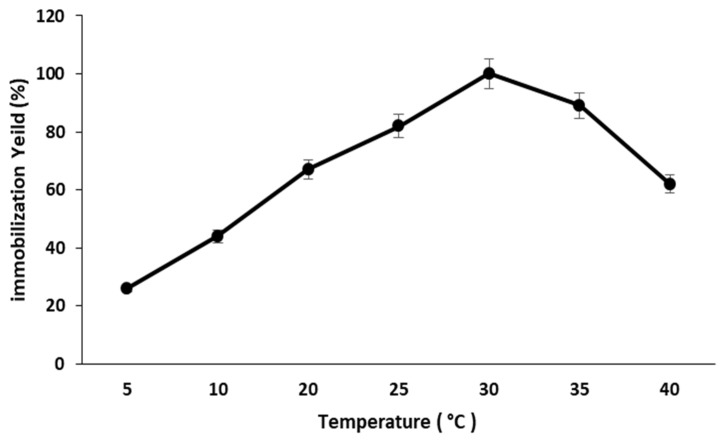
Effect of temperature on the immobilization of pectinase on magnetic iron oxide nanocarrier.

The impact of temperature on pectinase immobilization on magnetic iron oxide nanocarrier was investigated by performing the immobilization technique at various temperatures ranging from 5 °C to 40 °C. The immobilization yield of nanocarrier-immobilized pectinase increased with temperature, with the maximum immobilization yield achieved at 30 °C. The immobilization yield of pectinase was reduced as the temperature increase (Figure 5).

### 2.3. Characterization of Immobilized Pectinase Magnetic Iron Oxide Nanocarrier with Comparison of Free Pectinase

#### 2.3.1. Effect of Time on the Catalytic Activity of Magnetic Iron Oxide Nanocarrier Immobilized and Free Pectinase

**Figure 6:**
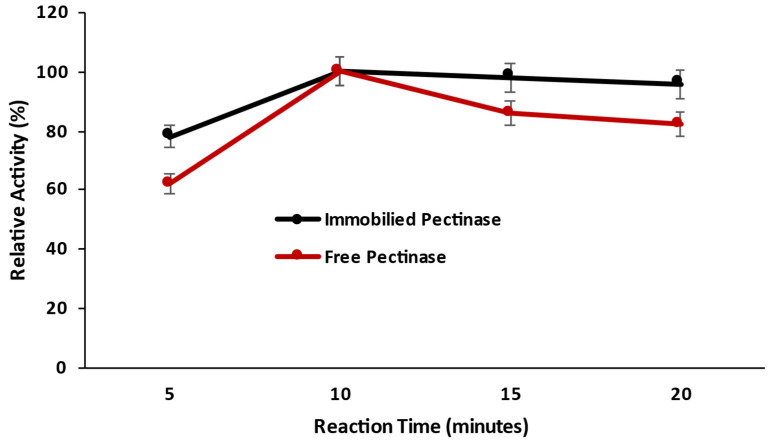
Effect of time on the catalytic activity of pectinase immobilized magnetic iron oxide nanocarrier.

The effect of incubation time on the enzymatic response of immobilized pectinase was studied by running the enzyme for different time periods ranging from 5.0 to 60 min and comparing it to free pectinase. Immobilization had no effect on the response of time pectinase, and both nanocarrier-immobilized pectinase and free pectinase exhibited maximal relative activity after 10 min of reaction time (Figure 6). The magnetic iron oxide nanocarrier had no effect on the structure of pectinase, and immobilized pectinase required the same reaction time for catalytic activity.

#### 2.3.2. Effect of Temperature on the Catalytic Activity of Magnetic Iron Oxide Nanocarrier Immobilized and Free Pectinase

**Figure 7:**
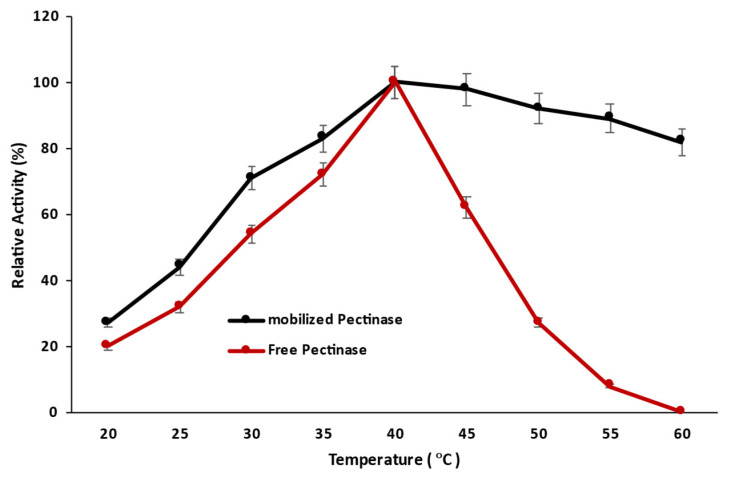
Effect of temperature on the catalytic activity of pectinase immobilized magnetic iron oxide nanocarrier.

The determination of the optimum temperature for maximum enzymatic activity is a critical step in investigating enzyme industrial feasibility; thus, the effect of temperature on the catalytic performance of iron oxide nanocarrier was determined by measuring the enzyme assay at different temperatures ranging from 20 to 60 °C with a comparison of free pectinase (Figure 7). The immobilization had no effect on the optimum temperature of pectinase, and both the immobilized and free enzymes performed optimally at the same temperature. However, magnetic iron oxide nanocarrier immobilization increased pectinase temperature resistance, and pectinase retained more than 80% of its catalytic activity at 60 °C, compared to free pectinase, which lost all activity. This rising temperature for maximal enzyme activity may be related to the physical limitations of pectinase inside the microenvironment of calcium alginate beads, as well as the higher activation energy necessary for the enzyme to diffuse into the substrate binding site [20]. However, the change in temperature for maximal enzyme activity to a higher level suggested that the support employed for enzyme immobilization could strengthen the conformational rigidity of the enzyme and protect it against denaturation at higher temperatures. It has previously been reported that immobilizing pectinase within calcium alginate beads by covalent bonding using glutaraldehyde as a crosslinking agent did not modify the optimal temperature and that both free and immobilized pectinase exhibited maximal activity at the same temperature [9]. It was also observed that following entrapment in a polyvinyl alcohol sponge, the optimal temperature of pectinase for maximal enzymatic activity was moved from 40 °C to 50 °C [21].

#### 2.3.3. Effect of pH on the Catalytic Activity of Magnetic Iron Oxide Nanocarrier Immobilized and Free Pectinase

**Figure 8:**
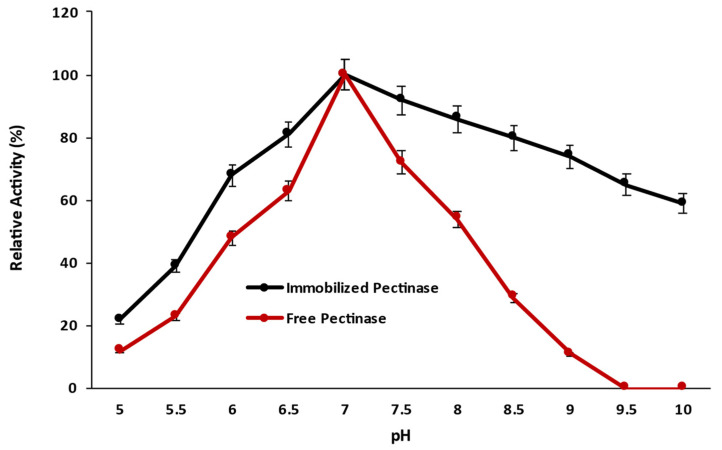
Effect of pH on the catalytic activity of pectinase immobilized magnetic iron oxide nanocarrier.

The influence of pH on the catalytic activity of magnetic iron oxide nanocarrier immobilized pectinase was studied by performing the enzyme assay at different reaction pHs spanning from pH 5 to pH 10 and comparing it to free pectinase (Figure 8). The immobilization had no effect on the optimal pH, and pectinase operated best in both immobilized and free forms at pH 7.0. The influence of pH on free pectinase was greater than that on immobilized pectinase, and magnetic iron oxide nanocarrier immobilization expanded the optimal pH range, with immobilized pectinase showing better relative activity at acidic and alkaline pH ranges than free pectinase. The pH profile of the enzyme revealed that covalent binding of the enzyme to a magnetic iron oxide nanocarrier increased resistance to a variety of pH ranges, and immobilized pectinase exhibited higher relative activity in a variety of pH ranges than free pectinase. Immobilized pectinase retained more than 50% of its relative activities at pH 10 when compared to free pectinase, which showed no activity at pH 9.5. Previously, it was observed that immobilization had no effect on pectinase’s optimal pH and that pectinase preserved its optimal pH for maximal enzymatic activity before and after immobilization but that the range of relative activity was expanded to some extent after immobilization [4,10,22].

#### 2.3.4. Thermal Stability of Magnetic Iron Oxide Nanocarrier Immobilized and Free Pectinase

**Figure 9:**
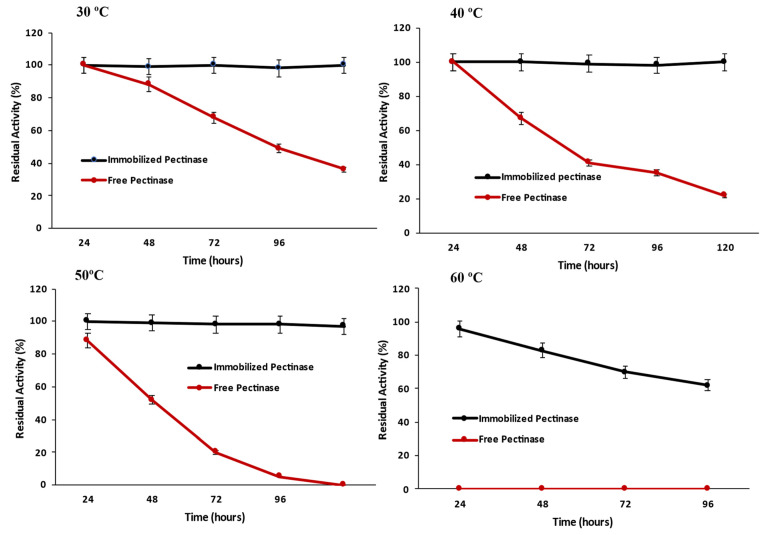
Thermal stability of pectinase immobilized magnetic iron oxide nanocarrier against different temperature.

The thermal stability of magnetic iron oxide nanocarrier-immobilized pectinase against different temperatures was investigated by pre-incubating the immobilized and free pectinase at various temperatures ranging from 25 °C to 60 °C and measuring enzyme activity every 24 h (Figure 9). It was observed that immobilized pectinase inactivated more slowly than free pectinase with temperature rise and retained 100% of its initial activity after 120 h at 30 °C and 40 °C, respectively, but free enzyme only retained 40% and 30% of activity at the same temperatures. The magnetic iron oxide nanocarrier immobilization increased pectinase thermal stability, with immobilized pectinase maintaining more than 90% residual activity at 60 °C after 120 h, while free pectinase lost all activity at the same temperature and time. The enhanced thermal stability of enzymes following immobilization has been attributed to the stabilizing impact of the support matrix, which limits the enzyme’s conformational changes in terms of thermal denaturation [23]. However, after 120 h, the immobilized pectinase preserved 60% of its original activity. Enzymatic activity was reduced at specific temperatures owing to the denaturation impact of these temperatures. It has been shown that at 65 °C, both free and immobilized pectinases lose their entire activity [10].

#### 2.3.5. Kinetic Parameters of Magnetic Iron Oxide Nanocarrier Immobilized and Free Pectinase

The kinetic parameters K_m_ and V_max_ were calculated by repeating the enzyme assay under standard assay conditions with varied concentrations of citrus pectin. The K_m_ and V_max_ values were determined by plotting 1/[V] versus 1/[S] (Table 1). [24]. After immobilization on a magnetic iron oxide nanocarrier, the K_m_ value of pectinase was slightly increased from 1.017 to 1.024 mg mL^−1^, and the V_max_ value was reduced from 23,800 to 22,600 µM min^−1^. It was previously reported that the K_m_ value of *A. niger* pectinase was enhanced from 2.0 to 3.2 mg mL^−1^, and the V_max_ value was decreased from 5.0 to 3.8 mg mL^−1^ following immobilization in comparison to the free enzyme [20]. It was also observed that employing co-precipitated silica coated surface modified magnetic nanoparticles as a coupling agent increased the affinity of pectinase toward its substrate owing to the expansion of accessible active sites across the support’s small and nanoporous surface [25].

#### 2.3.6. Reusability of Magnetic Iron Oxide Nanocarrier Immobilized Pectinase

**Figure 10:**
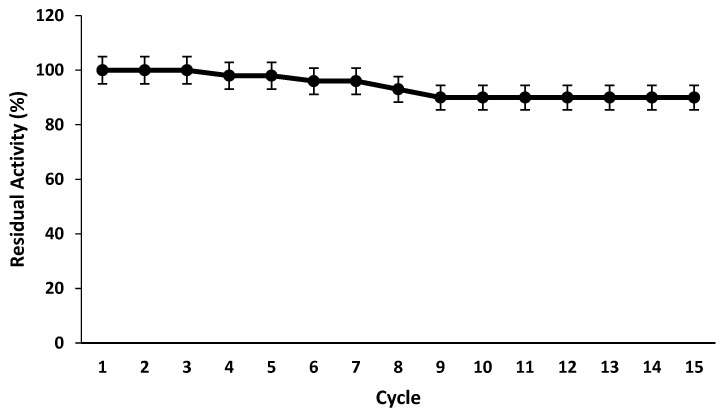
Reusability of pectinase immobilized magnetic iron oxide nanocarrier in reaction cycles.

Reusability is one of the most important characteristics of immobilized enzymes for its financial feasibility in industrial bioprocessing fixed systems [26]. As a result, the reusability of magnetic iron oxide nanocarrier immobilized pectinase was determined under standard assay conditions in 15 repetitive cycles using citrus pectin as substrate, and then the magnetic iron oxide nanocarrier immobilized pectinase was washed with denoised water, and fresh substrate was added for the next reaction cycle (Figure 10). Even after 15 reaction cycles, the magnetic iron oxide nanocarrier that immobilized pectinase retained about 90% of its initial activity. The operational stability of pectinase was increased after immobilization on nanocarrier, which might be attributed to covalent binding and the large surface area of magnetic iron oxide nanocarrier, which improved the stability of immobilized pectinase over simple entrapment [4]. However, the operational stability values of this experiment are higher than those previously reported for pectinase immobilization under the same conditions using the same support [5].

## 3. Material and Methods

### 3.1. Material

All of the chemicals used in the study were of analytical grade and were obtained from BDH (VWR, Poole, UK), Sigma (Burlington, MA, USA), Scharleu (Cham, Germany), and Merck (Da, Germany). All glassware was cleaned by soaking it in detergent and then scouring it with a bristle brush. Before use, it was thoroughly washed with tap water, rinsed with deionized water, and oven dried.

### 3.2. Microorganism and Pectinase Production

*Bacillus subtilis*, a previously obtained bacterial strain, was employed for pectinase synthesis using the submerged fermentation technique. The pectinase was partially purified from culture media by centrifugation at 10,000× *g* for 15 min at 4 °C, followed by precipitation with ammonium sulfate at 50% saturation. The precipitates were separated by centrifugation at 10,000 rpm for 15 min, then re-dissolved in glycine-NaOH and dialyzed for 3 h against the same buffer. For immobilization and catalytic characterization, dialyzed pectinase was used.

### 3.3. Synthesis of Iron Oxide Nanoparticles

Using the co-precipitation method, ferrous chloride tetrahydrate (FeCl_3_·6H_2_O) and ferric chloride hexahydrate (FeCl_3_·6H_2_O) were combined in a 1:2 molar ratio [27]. Continuous stirring of the two solutions was followed by the dropwise addition of ammonia solution to produce the basic medium, which led to the formation of black precipitates. The mixture was stirred vigorously for 24 h at 70 °C. After the precipitates cooled to room temperature, they were filtered and washed with deionized water and ethanol (3×). The product was dried at 100 °C under vacuum and calcinated at 600 °C for two hours at a rate of 20 °C every ten minutes. The final mixture was cooled to room temperature and held under a vacuum for enzyme immobilization characterization and application [28,29,30].

### 3.4. Scanning Electron Microscopy (SEM)

Scanning electron microscopy was performed using JSM Jeol 6380A (Japan) scanning electron microscope to analyze the surface topography and size of magnetic iron oxide nanoparticles.

### 3.5. Energy Dispersive Electron Microscopy (EDEX)

Energy Dispersive Electron Microscopy (EDEX) analysis was carried out by standard addition method with an accelerating voltage of 20 KeV to determine the elemental composition of magnetic iron oxide nanoparticles using.

### 3.6. Immobilization of Pectinase on Magnetic Iron Oxide Nanoparticles

For cross-linking, 20 mg of magnetic iron oxide nanoparticles were added to 5 mL of a 5% glutaraldehyde solution and incubated at 4 °C for 3 h. The magnetic iron nanoparticles were then extracted magnetically from the solution and washed with a buffer (50 mM sodium phosphate buffer, pH 7.5) to remove the unbound glutaraldehyde. The magnetic iron nanoparticles were then incubated with 1 mL of pectinase at 4 °C for three hours. Then, the pectinase-immobilized magnetic iron nanoparticles were collected using an external magnetic field, washed with buffer (50 mM sodium phosphate, pH 7.5), and kept at 4 °C for the activity assay.

Immobilization yield was calculated at each step using following formula:

$$\mathrm{Immobilization}\;\mathrm{Yield}=\frac{\mathrm{Total}\;\mathrm{activity}\;\mathrm{of}\;\mathrm{Immobilized}\;\mathrm{enzyme}}{\mathrm{Total}\;\mathrm{activity}\;\mathrm{of}\;\mathrm{soluble}\;\mathrm{enzyme}}\times 100$$

### 3.7. Enzyme Assay

Using the DNS approach, the enzyme activity of free and iron oxide-immobilized pectinase was determined by estimating the amount of reducing sugar. The substrate was citrus pectin and the standard was galacturonic acid [31] under optimum reaction conditions (pH 7, 40 °C, and 10 min). Under standard assay conditions, one unit (IU) of pectinase is defined as “the quantity of enzyme necessary to produce 1 μmol of galacturonic acid”.

### 3.8. Determination of Total Protein

Lowry’s technique with BSA (bovine serum albumin) as a standard was used to determine the total protein [32]. The amount of immobilized protein was determined by subtracting the amount of protein remaining in the solution at the end of the immobilization procedure and in the washing solutions from the total amount of protein used for immobilization.

### 3.9. Optimization of Immobilization Parameters of Pectinase on Magnetic Iron Nanoparticles

#### 3.9.1. Effect of Crosslinking Concentration

The effect of glutaraldehyde concentration on the immobilization of pectinase in terms of retention of maximum enzymatic activity was determined using various concentrations of glutaraldehyde ranging from 0.5% to 5.0% during the immobilization procedure keeping the other parameters constant.

#### 3.9.2. Effect of Enzyme Concentration

During the immobilization procedure, various concentrations of enzyme solution ranging from 500 to 5000 IU/mg were used to determine the impact of pectinase concentration on pectinase immobility.

#### 3.9.3. Effect of Crosslinking Time

In order to determine the effect of crosslinking time on the immobilization of pectinase on nanocarriers, the glutaraldehyde solution was coupled with nanocarriers for 30 to 300 min.

#### 3.9.4. Effect of Immobilization Temperature

Analyzing the influence of temperature on the immobilization of pectinase on magnetic iron oxide nanocarriers, the immobilization method was carried out at temperatures ranging from 5 °C to 40 °C.

### 3.10. Characterization of Immobilized Pectinase Magnetic Iron Oxide Nanocarrier with Comparison of Free Pectinase

#### 3.10.1. Effect of Time on the Catalytic Activity of Magnetic Iron Oxide Nanocarrier Immobilized and Free Pectinase

The effect of the incubation period on the enzymatic reaction of immobilized pectinase was analyzed by performing the enzymatic reaction for various time periods ranging from 5.0 to 60 min and comparing the results to those obtained with free pectinase.

#### 3.10.2. Effect of Temperature on the Catalytic Activity of Magnetic Iron Oxide Nanocarrier Immobilized and Free Pectinase

The effect of temperature on the catalytic performance of iron oxide nanocarriers was investigated by evaluating the enzyme assay at temperatures ranging from 20 to 50 °C and comparing the results to those obtained with free pectinase.

#### 3.10.3. Effect of pH on the Catalytic Activity of Magnetic Iron Oxide Nanocarrier Immobilized and Free Pectinase

The effect of pH on the catalytic activity of magnetic iron oxide nanocarrier immobilized pectinase was determined by performing the enzyme assay in different reaction pH ranging from pH 5 to pH 10 and comparing the results to the catalytic activity of free pectinase.

#### 3.10.4. Thermal Stability of Magnetic Iron Oxide Nanocarrier Immobilized and Free Pectinase

The thermal stability of magnetic iron oxide nanocarrier immobilized pectinase against various temperatures with the comparison of the free enzyme was determined by pre-incubating the immobilized and free pectinase at various temperatures ranging from 25 °C to 60 °C and every 24 h the enzyme activity was measured.

The thermal stability of magnetic iron oxide nanocarrier-immobilized pectinase against different temperatures was determined by pre-incubating the immobilized and free pectinase at different temperatures ranging from 25 to 60 °C and measuring the enzyme activity every 24 h.

#### 3.10.5. Kinetic Parameters of Magnetic Iron Oxide Nanocarrier Immobilized and Free Pectinase

Under standard assay conditions, the kinetic parameters K_m_ and V_max_ were estimated by performing an enzyme assay with different concentrations of citrus pectin. The K_m_ and V_max_ values were determined by plotting 1/[V] versus 1/[S] [24].

#### 3.10.6. Reusability of Magnetic Iron Oxide Nanocarrier Immobilized Pectinase

The reusability of magnetic iron oxide nanocarrier immobilized pectinase was determined under standard assay conditions using citrus pectin as substrate in 15 repetitive cycles and after the magnetic iron oxide nanocarrier, immobilized pectinase was washed with denoised water, and fresh substrate was added for next reaction cycle.

## 4. Conclusions

Pectinase was generated from B. subtilis and immobilized on magnetic iron oxide nanoparticles using glutaraldehyde as a crosslinking agent in the current study to improve its industrial uses and operational stability. Magnetic iron oxide nanoparticles were prepared using the co-precipitation process and analyzed using scanning electron microscopy (SEM) and energy dispersive electron microscopy (EDEM) (EDAX). When compared to free pectinase, magnetic iron oxide nanocarrier improved pectinase catalytic properties, and immobilized pectinase demonstrated higher relative activity against pH and temperature variations. The thermal stability of pectinase was increased after immobilization of iron oxide nanocarrier, and immobilized pectinase retained more than 90% of its original activity at 50 °C after 120 h of pre-incubation. After 10 batches of reaction cycles, the magnetic iron oxide nanocarrier immobilized pectinase and preserved 100% of its original activity. The immobilization of pectinase in magnetic iron oxide nanocarrier using glutaraldehyde as a crosslinking agent improved its operational stability and catalytic characteristics.

## Figures and Tables

**Table 1:** Kinetic Parameters of magnetic iron oxide nanocarrier immobilized and free pectinase.

Enzyme	K_m_ (mg mL^−1^ min^−1^)	V_max_ (µM min^−1^)
Free Pectinase	1.017	23,800
Magnetic Iron Oxide Immobilized Pectinase	1.024	22,600

## Data Availability

Data available on request due to restrictions (Privacy).
